# The Use of PRV-Bartha to Define Premotor Inputs to Lumbar Motoneurons in the Neonatal Spinal Cord of the Mouse

**DOI:** 10.1371/journal.pone.0011743

**Published:** 2010-07-23

**Authors:** Ksenija Jovanovic, Angel M. Pastor, Michael J. O'Donovan

**Affiliations:** 1 Developmental Neurobiology Section, National Institute of Neurological Disorders and Stroke, National Institutes of Health, Bethesda, Maryland, United States of America; 2 Laboratorio Reparación Neural y Biomateriales, Hospital Nacional de Parapléjicos, Toledo, Spain; 3 Departamento de Fisiología y Zoología, Facultad de Biología, Universidad de Sevilla, Sevilla, Spain; Emory University, United States of America

## Abstract

**Background:**

The neonatal mouse has become a model system for studying the locomotor function of the lumbar spinal cord. However, information about the synaptic connectivity within the governing neural network remains scarce. A neurotropic pseudorabies virus (PRV) Bartha has been used to map neuronal connectivity in other parts of the nervous system, due to its ability to travel trans-neuronally. Its use in spinal circuits regulating locomotion has been limited and no study has defined the time course of labelling for neurons known to project monosynaptically to motoneurons.

**Methodology/Principal Findings:**

Here we investigated the ability of PRV Bartha, expressing green and/or red fluorescence, to label spinal neurons projecting monosynaptically to motoneurons of two principal hindlimb muscles, the tibialis anterior (TA) and gastrocnemius (GC). As revealed by combined immunocytochemistry and confocal microscopy, 24–32 h after the viral muscle injection the label was restricted to the motoneuron pool while at 32–40 h the fluorescence was seen in interneurons throughout the medial and lateral ventral grey matter. Two classes of ipsilateral interneurons known to project monosynaptically to motoneurons (Renshaw cells and cells of origin of C-terminals) were consistently labeled at 40 h post-injection but also a group in the ventral grey matter contralaterally. Our results suggest that the labeling of last order interneurons occurred 8–12 h after motoneuron labeling and we presume this is the time taken by the virus to cross one synapse, to travel retrogradely and to replicate in the labeled cells.

**Conclusions/Significance:**

The study establishes the time window for virally - labelling monosynaptic projections to lumbar motoneurons following viral injection into hindlimb muscles. Moreover, it provides a good foundation for intracellular targeting of the labeled neurons in future physiological studies and better understanding the functional organization of the lumbar neural networks.

## Introduction

The mouse lumbar spinal cord has become a model system in which to study locomotor function because the underlying neural networks can be activated chemically or electrically and studied in the absence of an instructive input drive. Recently, different classes of spinal interneurons have been identified by their expression of transcription factors [see 1–3 for reviews] and deleted or silenced genetically to establish their role in locomotion [4]–[6]. However, methods for identifying the synaptic connections within the network remain elusive. One approach to this problem is to use neurotropic viruses that can propagate trans-synaptically [7]–[13]. One of them, PRV Bartha is an attenuated live vaccine strain that propagates retrogradely through chains of functionally connected neurons [14] The Bartha strain is significantly less cytopathic than the other members of the family which allows for a prolonged incubation time in the host and greater infection efficacy [15]. Furthermore, the labeling does not require experimental activation of neuronal circuits and can be extensive, involving second order or even higher order interneurons [10]. Although the virus has been injected into a number of peripheral sites, including the diaphragm [16], the gastrointestinal tract [17] the prostate [18], and shown to propagate into the brain regions known to innervate these peripheral targets only a few studies have used it to map neurons in the spinal cord [19]–[21] or to identify the projections of some genetically labeled interneurons to motoneurons [22]. However, no study has systematically investigated the time course of the viral propagation into spinal motoneurons and/or interneurons and established its ability to label neurons - such as Renshaw cells - that are known to project monosynaptically to motoneurons. This is important because the time of viral transfer may vary in different groups of neurons. Moreover, because the virus can label neurons through second and third order connections, defining the time required to label first order monosynaptic projections is crucial to establish if unknown connections are monosynaptic.

In the present work, we used the PRV Bartha coupled to green and red fluorescent proteins to define the time course of viral labeling following injection of the virus into the tibialis anterior and, on some occasions, the gastrocnemius muscle in the neonatal mouse. Our study provides a new insight into the transneuronal viral labeling showing that the virus spreads from motoneurons to last-order interneurons within 8–12 h, and that within 40 h postinjection successfully labels interneurons known to project monosynaptically to motoneurons. Furthermore, within the same time frame, it reveals the existence of some neuronal classes whose relationship to motoneurons and possible role in locomotor network remains to be defined. A preliminary report of this work has been published in abstract form [23].

## Materials and Methods

### The viral vector

We used two isogenic recombinants of an attenuated pseudorabies virus strain Bartha (PRV Bartha) that express enhanced green fluorescent protein (PRV152) or the monomeric red fluorescent protein PRV614 [24]. The viral recombinants were harvested from pig kidney cell cultures at titers 5.6×10^8^ and 4.8×10^8^ plaque forming units (pfu/µl), respectively and generously provided by Dr. Bruce Banfield (Queen's University, Canada; see [12]) and Dr. Botond Rosca (Neural Circuit Laboratories, Friedrich Miescher Institute for Biomedical research, Basel, Switzerland). Viral stocks were aliquoted at 100 µl volumes in 1 ml vials and stored at −80°C until a few minutes before use. Detailed information comprising a gene image map for the PRV genome can be found at the Los Alamos website: http://oralgen.lanl.gov/oralgen/bacteria/prv/.

### Surgery and viral injections

Experiments were performed on approximately one week old (postnatal day P7–P8) Swiss-Webster mice (n = 50). The preparation, surgical procedures and animal care were carried out under Bio Safety level 2 conditions (BSL2), in accordance with the National Institute of Health guidelines and approved by the NINDS Animal Care and Use committee.

**Figure 1:**
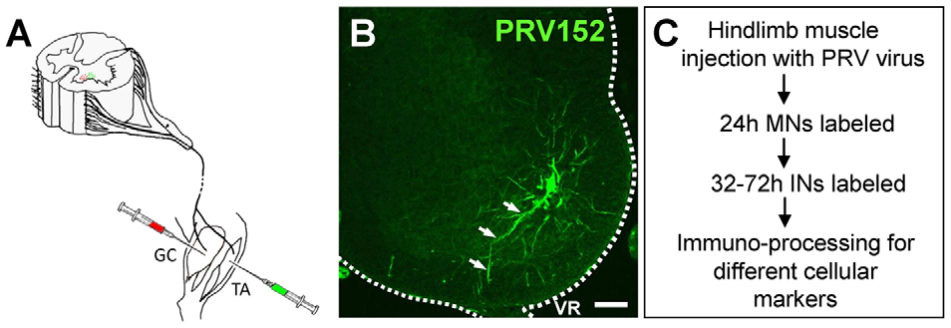
Experimental Paradigm. **A**) Schematic representation of a spinal cord cross-section and the hindlimb of the mouse. At the age of P7–8, the tibialis anterior (TA), alone or together with the gastrocnemius muscles (GC), was injected with recombinant strains of PRV Bartha virus conjugated to either green (PRV152 - EGFP) or red (PRV614 - RFP) fluorescent protein. **B**) Low magnification confocal image of the lumbar spinal cord illustrating one TA motoneuron retrogradely labelled with the neurotropic PRV152-EGFP 24 h after the injection. Following different post-injection times, viral replication leads to a strong amplification of the reporter protein in the infected neurons revealing their detailed morphology. The labelled axon (arrows) projects towards the ventral root (VR). Scale bar 100 µm. **C**) Overview of the intramuscular PRV labelling and processing protocol.

Animals were anesthetized with Halothane under aseptic conditions and small skin incisions, exposing the tibialis anterior (TA) and gastrocnemius muscles (GC, medial and lateral head), were made under a dissecting microscope. Injections of PRV152-EGFP and/or PRV614-RFP were made in multiple sites in both crural and costal regions of the muscle (Fig. 1A) using 10 µl Hamilton syringe equipped with a 33 gauge needle (Hamilton; Reno, NV). Each muscle was injected either with the virus alone or in combination with a classical retrograde marker such as tetramethylrhodamine dextran (TMRD, 10 mM in ACSF, MW 3000; Molecular Probes; Eugene OR) or wheat germ agglutinin (WGA-Texas Red conjugate; 10% solution in ACSF, Molecular Probes; Eugene, OR). However, when the virus was injected into a muscle together with another retrograde marker, the viral labeling was often lower and less robust than that obtained with the retrograde label (data not shown). To circumvent this problem, and to evaluate the efficacy of the virus as a neuronal marker, we compared the labeling pattern it produced in motoneurons (MNs) with the labeling produced by the retrograde label Fluorogold (FG; Fluorochrome, Denver, CO) and immunostaining against choline acetyltransferase (ChAT). Albeit through different mechanisms, both markers produce consistent and widespread labeling of somatic MNs allowing for a direct comparison with the viral labeling pattern. Thus, in some experiments animals were injected with FG 24–48 h before the viral injection to produce global labeling of somatic motoneurons [25], [26]. The marker was first diluted in 0.9% physiological saline and administered intraperitoneally (IP, 50 µg/g).

Following different post-injection times (8–72 h), animals were re-anesthetized by Halothane and some of them decapitated, eviscerated and their spinal cords dissected out at room temperature in a chamber constantly superfused with cooled and oxygenated (5% CO_2_/95% O_2_) artificial cerebrospinal fluid (ACSF, composition in mM: 128.35 NaCl, 4KCl, 0.58 NaH_2_PO_4_*H2O, 21 NaHCO_3_, 1.5 CaCl_2 *_ H_2_O, 1MgSO_4*_ 7H_2_O and 30 D-glucose). Their spinal cords were then immersion-fixed in 4% paraformaldehyde (4% PFA/0.01M PBS) for 4 h, cryoprotected in 30% sucrose and cast frozen in O.C.T. (Tissue Tek, EMS, Hartfield, PA) for cutting on a Cryostat. However, due to the superior appearance of the tissue, the majority of animals were transcardially perfused with 4% paraformaldehyde (saline, followed with 4% PFA/0.01PBS) following terminal anesthesia. Lumbar spinal cords were then removed from the animal and postfixed in the same fixative for 4 h. They were subsequently rinsed in 0.01M PBS and embedded in warm agar (5% agar/PBS) until solidified. Serial sections (50 µm) were cut on a Vibratome Leica Microsystems Inc. and processed using floating immunocytochemistry. The spinal cords were prepared for cutting in one of two ways: either separate lumbar segments were cut into individual blocks and sectioned coronally or alternatively, the L2–L6 segments were cut in the horizontal plane. Sections were collected on gelatinized slides and the patterns of expression of GFP/RFP and immunolabeling were examined in serial, transverse or horizontal sections of the lumbar spinal cord.

### Antibodies and Immunostaining procedures

The primary antibodies used in this study were all purchased from Chemicon International (Temecula, CA; presently Millipore). The central projections of muscle proprioceptors were identified by their expression of calcium binding protein parvalbumin (PV) immunoreactivity using a mouse monoclonal antibody (1∶1000; MAB1572). This antibody recognizes a protein of 12 kD by immunoblot and it is directed against an epitope at the first Ca^+2^-binding protein, specifically staining the Ca^+2^-bound form of parvalbumin (manufacturer's information). Another calcium binding protein calbindin-D28K (CB) has been shown to be a good marker for Renshaw cells in the ventral spinal cord [27]–[29]. For this reason, we used a rabbit polyclonal antibody (1∶500, AB1778) to immunolabel these cells. To immunostain MNs and other cholinergic neurons we used a goat polyclonal antibody specifically directed against choline acetyltransferase (ChAT; 1∶100, AB144P), which is abundant in these neurons. The pattern of staining with these antibodies was similar to that reported in previous studies for parvalbumin [30], [31], calbindin [27]–[29] and choline acetyltransferase [32], [33].

Serial Vibratome sections (50 µm) were immunolabeled free-floating while Cryostat sections (25 µm) were immunoreacted directly on glass slides. Initially, the tissue was rinsed several times in 0.01M PBS (6×10 min) and then submerged for 1 h in 10% normal donkey serum (diluted in 0.01 PBS with 0.1 Triton-X-100 (PBS-TX, Jackson ImmunoResearch, West Grove, PA) to block non-specific antibody binding. Subsequently, sections were incubated at 4°C overnight (or at room temperature for 8 h) in different combinations of primary antibodies previously diluted in PBS-TX. Following incubation with the primary antibodies, sections were rinsed in PBS-TX (6×10 min) and reacted for 2 h with secondary antibodies conjugated to different fluorochromes (FITC, Cy5, TRITC) and designed for multiple labeling paradigms (Jackson ImmunoResearch, West Grove, Pasadena, CA). Secondary antibodies were used at working dilutions of 1∶50 to 1∶100 and all reactions involving them were carried out at room temperature. After one more series of rinses (6×10 min) in PBS, sections were mounted on slides and cover-slipped in ProLong Gold anti-fading agent (Molecular Probes, Eugene, OR). Tissue sections in which primary or secondary antibodies were omitted were kept as a control to detect background due to autofluorescence and/or none specific binding of the secondary antibodies.

### Analysis

The location and morphology of retrogradely labeled neurons (containing EGFP or RFP) were examined in transverse and horizontal sections and images acquired using a three channel 510 META confocal microscope (Carl Zeiss, Germany) or a Leica SP5 confocal microscope with a resonant scanner (Leica Microsystems, Germany). Both microscopes were equipped with Argon, HeNe and UV (diode 405 nm) lasers while the Leica SP5 also contained a diode-pumped solid state laser (DPSS). To visualize neuronal labeling by PRV152-EGFP, tissue sections were excited by using Argon laser (488 nm) and images collected in the emission range 495–550 nm while the tissue containing PRV614-RFP was excited by using DPSS laser (561 nm) and images collected in the emission range 566–630 nm. Tissue immunolabeled with Cy5 was excited using a HeNe laser (633 nm) and the resulting images collected in the emission range 640–740 nm. Depending on the combination of fluorescent probes we used, detection parameters were optimized in each scanning session and sequential scanning performed to avoid fluorescence crosstalk between the channels. All images presented in this study are two-dimensional projections obtained from serial optical sections (z-stacks) with image contrast and brightness adjusted in Adobe Photo shop 7.0 (Adobe Systems Inc., San Jose, California; Mac edition) to optimize the visibility of neurites and/or somata of small neurons. As a result, somata of large neurons were occasionally color saturated.

Neuronal counts were performed on z-stacks of confocal optical sections obtained from lumbar spinal cord segments. Under all conditions, only those cells in which a clear, well defined somatic profile could be seen were counted. If a cell was present in consecutive sections, only the section that provided the largest soma profile was used. Neuronal counts are expressed as mean ± SD. Cell size was measured using Image J (NIH, Bethesda, MA). The maximum and minimum diameters of cells and their surface area were measured and averaged for 22 to 141 motoneurons per post-injection time group. Comparisons between the groups were performed using one-way ANOVA followed by the Holm-Sidak method for post-hoc testing with significance level being set at p≤0.01. The statistical analysis was performed using commercial software (SigmaStat 9, Point Richmond, CA).

## Results

**Figure 2:**
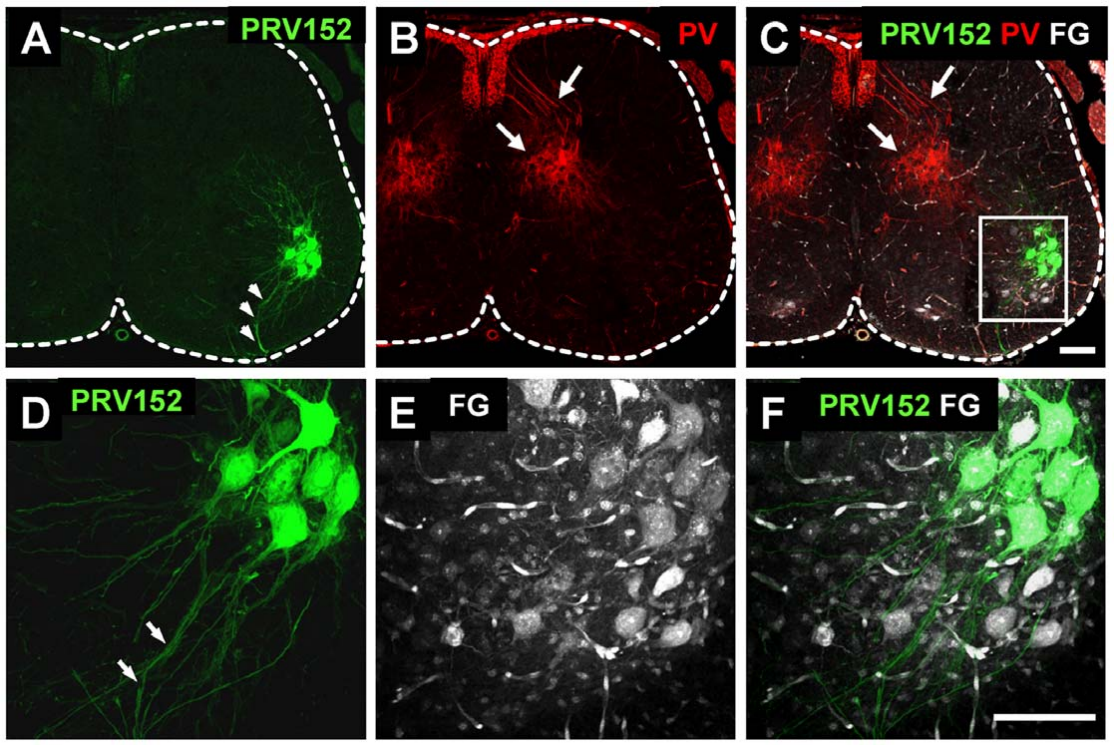
Viral labelling by muscle injection is restricted to the parent motor pool and is not detected in afferent fibers 28 hours after the muscle injection. **A–C**) Low magnification confocal images of a section from the 4^th^ lumbar segment (L4) obtained 28 h after the TA muscle was injected with PRV152-EGFP (**A**, green) and muscle afferents were labelled with parvalbumin (**B**, PV). **C**). The images in A and B have been merged with an image showing motoneurons retrogradely labelled by intra-peritoneal injection of Fluorogold (FG, white). Note that viral labelling is absent in the afferents and restricted to a subset of motoneurons in the known location of TA motoneuron pool. **D–E**). High magnification images of the boxed region in panel C, to show the overlap of the viral label (**D**) and the Fluorogold label (**E**, FG) in TA motoneurons. **F**). Merged image of D and E. Scale bars are 100 µm.

To distinguish between the labeling of motoneurons and other spinal neurons (hereafter referred to as interneurons) we compared the pattern of viral labeling with that of the motoneuron marker ChAT or the retrograde marker Fluorogold. This approach allowed us to establish when motoneurons first became labeled with the virus and when the first non-cholinergic interneurons were labeled. As shown in figures 1 and 2, 24–28 hours after the injection of the virus into the TA muscle, fluorescence was restricted to motoneurons in the location of the TA motor pool (Fig. 2A–D).

### Viral labeling of Motoneurons

Viral labeling was monitored at 4–12 h intervals, up to 3 days (72 h) following the intramuscular injections. Post-injection intervals shorter than 24 h did not result in viral labeling of any neurons. However, at 24–28 h following PRV152 injections into the TA muscle, green fluorescence (EGFP) conjugated to the viral vector was expressed only in motoneurons ipsilateral to the injection (Fig. 1B; 2A, C).

**Figure 3:**
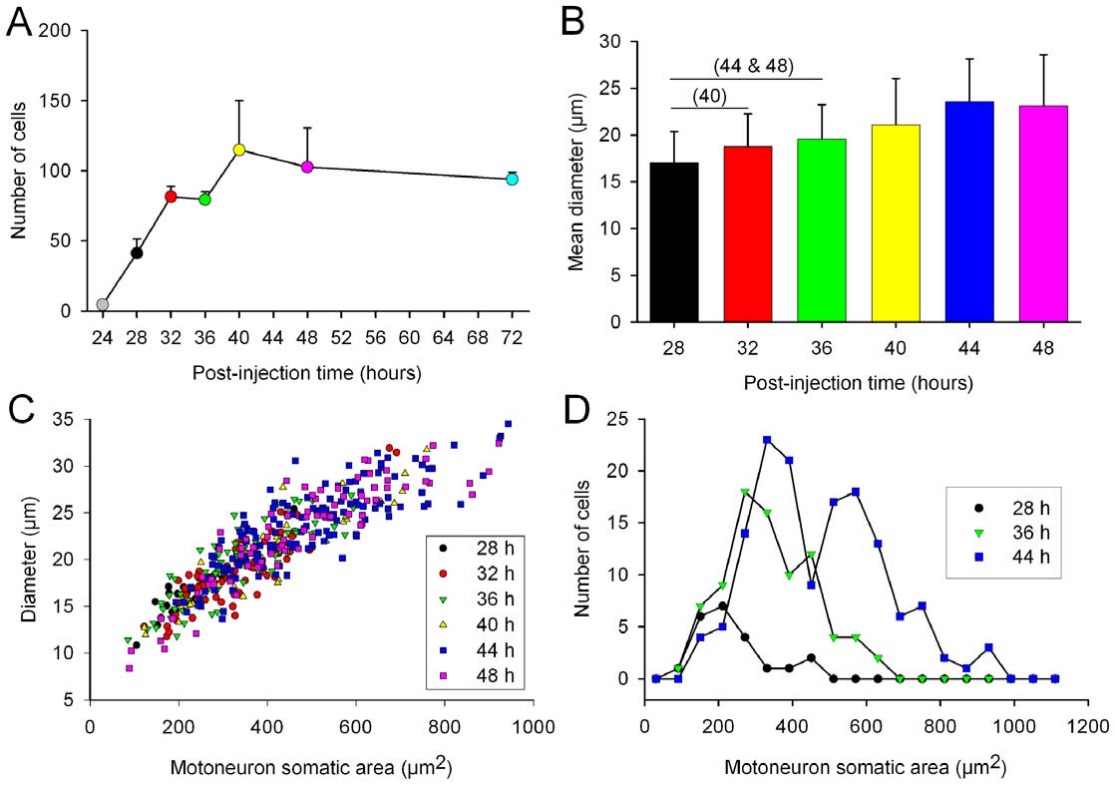
Change in the number of labelled motoneurons and their size with different survival times. **A**). Change in the number of labelled motoneurons as a function of post-injection time. Labelled motoneurons were first detected at 24–28 hours following muscle injection and their number stabilized at 40 h post-injection. The color code was assigned to different time points for clarity and maintained in all panels. **B**) Time course of changes in soma size as a function of post-injection time for animals injected at postnatal day 7. The means of the averaged diameters per post-injection time group (bars) and their standard deviations (error bars) are shown. The average diameter for each cell was calculated as the arithmetic mean of the maximum and minimum diameters. The number of cells per group was 22, 83, 83, 40, 141, 75, respectively for different post-injection time groups. The numeral 40 in parenthesis above the 28–32 h bar denotes a statistically significant difference in soma diameter between the group surviving for 40 hours with respect to those surviving for 28 and 32 hours. Similarly, the groups surviving for 44 and 48 hours had a larger mean averaged soma diameter than each of both the 28 and 32 hours groups (one-way ANOVA test, Holm-Sidak method for all pairwise multiple comparisons; p≤0.01 for single comparisons). **C**) Scatter plots of soma diameters versus motoneuronal somatic area for the indicated survival time groups. Note that the 40 and 48 hour groups exhibit a widespread distribution of soma sizes whereas the 28 and 32 hours survival groups are distributed within a narrower size range. **D**) Distribution of motoneuron soma sizes for three representative survival times. Note that small cells prevail at 28 hours post-injection while the distribution is broader at 44 hours.

Motoneuron cell bodies, ranging from 8–35 µm, were found in the L4 and L5 spinal segments, corresponding to the location of the TA motoneuron pool and their axons projected out of the adjacent ventral root (Fig. 1B; arrows Fig. 2A and D). Only a few motoneurons in the pool were labeled at this time (4.8±0.9) and the number of labeled motoneurons progressively increased over the next 8 h so that at 28 h the count was 41±10.1 before stabilizing between 32–40 h post-injection when it reached 81.5±7.5 (n = 2; Fig. 3A). The number of TA motoneurons at 72 h was 103±11.0 (n = 10) although this was not significantly different from the number at 40 h post-injection (ANOVA test; Fig. 3A), indicating that motoneurons were not killed by the virus. This number is lower than previous estimates of the number of TA motoneurons in the neonatal rat [34] and mouse [35], [36]. Dekkers and collaborators [36] reported 176 motoneurons while Tissenbaum and Parry [35] found 141 using retrograde labelling methods. It is possible that the lower number of TA motoneurons detected in the present work resulted from the limited amount of virus we injected into the TA muscle to minimize its spread to other muscles.

To establish if there was any preferential uptake of the virus into small or large motor axons we measured the size of motoneuron somata in animals that were injected at P7 with PRV152 injections and survived 28 to 48 h post-injection. With increasing survival time we observed an increase in the soma size as measured either by the mean averaged soma diameter (Fig. 3B; ANOVA test; P<0.01), the somatic area (Fig. 3C, D) or the soma perimeter (not shown). At early survival times (24–32 h post-injection) motoneurons with somata of approximately 15–20 µm in diameter (around 200–400 µm^2^) were predominant (Fig. 3B, 3C, respectively), while at times longer than 32 hours, in addition to these small cells, there was an increasing number of virally labeled somata of 25–30 µm diameter (500–700 µm^2^) and some even larger than 30 µm diameter (i.e. >800 µm^2^, Fig. 3C, 3D). We hypothesize that the cells that label first with the virus are γ-motoneurons because it has been shown that these cells comprise the majority of motoneurons with a surface area <500 µm^2^ [37].

We found no evidence that muscle afferent axons within the spinal cord expressed the virus. Tissue immunoprocessing for the calcium binding protein parvalbumin, which labels primary afferent fibers, up to 40 h after the virus injection into the muscle, revealed that the viral fluorescence was restricted to motoneurons and their axons (Fig. 2) and was not expressed in afferent fibers (Fig. 2B,C; arrows). This makes it unlikely that the viral labeling of motoneurons or interneurons was derived trans-synaptically from muscle afferents.

### Viral labeling of Interneurons

**Figure 4:**
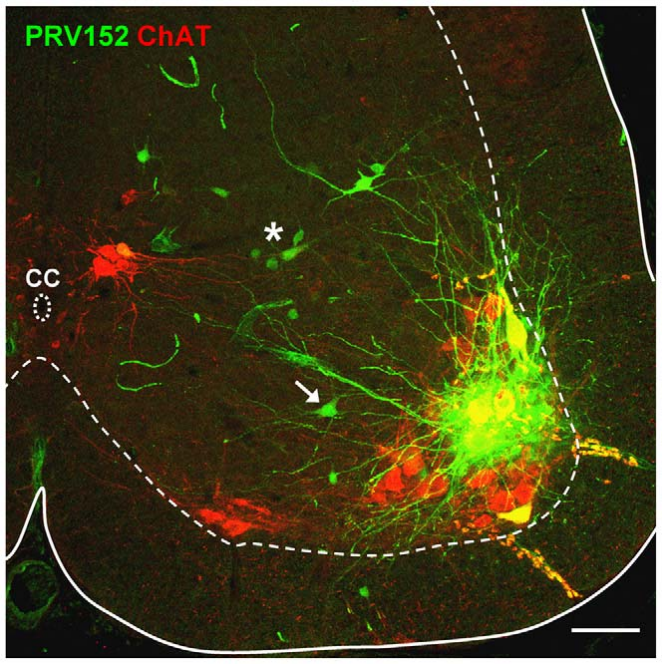
Different types of interneuron can be visualized by viral trans-synaptic propagation 36 h post-injection of PRV152-EGFP into the TA muscle. **A**) Confocal image of a transverse section in the L4 lumbar spinal cord obtained from a P7 pup in which PRV152 (green) was administered intramuscularly and spinal cord tissue immunoprocessed for ChAT (red) 36 h post-injection. Somatic motoneurons and cells near the central canal (cc) are ChAT-positive while large somata motoneurons in the TA pool are co-labeled with the virus (green) and ChAT (red). Different virally labelled interneurons are visible dorsal and medial to the MN pool. Some of them had small, unipolar somata (asterisk) while larger ones had multipolar somata suggestive of Ia interneurons (arrow). Scale bar 100 µm.

The first labeled interneurons were encountered at 32 h in the spinal cord ipsilateral to the virus injection. These early labeled cells were located in areas slightly dorsal and medial to the TA MN pool but did not appear in significant numbers until 36 h post-injection (Fig. 4). Between 36–40 h the number of virally labeled interneurons increased and they were also found dorsal, ventral and medial to the motoneuron pool. These were usually cells with simple unipolar somata with a diameter of up to 20 µm. However, we also observed some larger cells with more complex multipolar somata located dorsal and medial to the MN pool. Given their size and location, these cells may have been Ia inhibitory interneurons (arrow Fig. 4; see also [29]) that are known to project monosynaptically to motoneurons.

**Figure 5:**
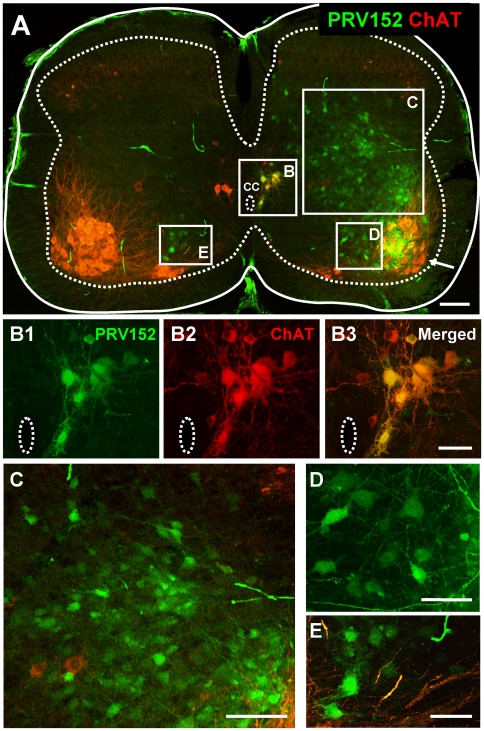
Distribution of virally labelled neurons at 40 h post-injection of the TA muscle with PRV152. **A**) Low magnification confocal image of a transverse section from the L4 segment obtained from a P8 mouse in which TA muscle was injected with PRV152 (green) and tissue subsequently immunoprocessed for ChAT (red) to differentiate motoneurons and other cholinergic neurons from interneurons. On the ipsilateral side, virally labeled neurons were found near the central canal (cc, box B) as well as throughout the central grey matter, dorsal (rectangle C) and medial (rectangle D) to the MN pool (arrow). Somatic motoneurons and cells near the central canal are ChAT-immunorective while motoneurons in the TA pool are co-labeled with the virus (green) and ChAT (red). At this post-injection time virally labelled cells are also found on the contralateral side (box E). Scale bar 100 µm. **B1–B3**) High magnification, split channel images of the corresponding boxed area in panel A, illustrate a cluster of virally labeled (green) neurons near the central canal (CC) that also co-express ChAT. Scale bar 50 µm. **C,** **D** and **E** are high magnification images of the regions indicated with corresponding boxes in panel A. Scale bars 100 µm for C and 50 µm for D and E.

By a survival time of 40 h, the number of labeled interneurons in the ipsilateral spinal cord significantly increased, particularly in laminae V–VII (Fig. 5). The virus expressing neurons were found throughout the ipsilateral grey matter (Fig. 5A, C) and the first contralateral interneurons to express the virus were observed at this time (Fig. 5A, E). Cholinergic neurons near the central canal (Fig. 5A, B) were also prominently labeled at 40 hr (Fig. 5B). The virally labeled cholinergic neurons near the central canal are not autonomic pre-ganglionic neurons (which are not present in lumbar segments 4 and 5) and are probably a recently identified class of cholinergic interneuron that are believed to be the origin of C-boutons on motoneurons [38], [39].

**Figure 6:**
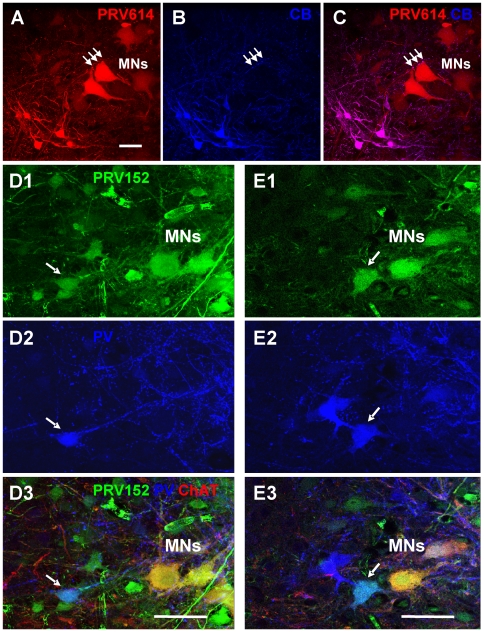
Trans-synaptic labeling of putative Renshaw cells (A–C) and Ia inhibitory interneurons (D, E) at 40 h post-injection. **A**) High magnification confocal image of the ventrolateral part of the L4 spinal segment (P8 animal) showing motoneurons (MNs) and smaller interneurons labelled with PRV614-RFP (red virus) following the injection of the virus into the tibialis anterior (TA) and gastrocnemius muscle (GC, medial and lateral head). **B**) The labelled interneurons are calbindin-immunoreactive and are therefore putative Renshaw cells. **C**) Merged image of A and B showing magenta labelled putative Renshaw cells. Note also the presence of doubly labelled putative synaptic swellings apposed to the motoneuron somata (small arrows). Scale bar 50 µm. **D, E**) High magnification confocal images of a lumbar L4 spinal section obtained from a P8 pup injected with the PRV152 (green virus) into the TA and GC muscle 40 h earlier and the tissue subsequently immuno-processed for ChAT (red) and parvalbumin (PV; blue). Two sets of images showing virally labelled interneurons (PRV152, green, D1 and E1, arrows) colabeled with the calcium binding protein parvalbumin (PV, blue, D2 and E2) suggesting that they may have been Ia inhibitory interneurons. Each image was located at a different depth in the section. The last set of images (D3 and E3), shows the merged viral and parvalbumin labelling together with immunoreactivity for ChAT (red) to label motoneurons (MNs). Scale bars 50 µm.

To establish whether some of the earliest labeled interneurons made direct monosynaptic contacts with motoneurons we investigated the expression of the calcium binding proteins calbindin and parvalbumin which are known to label Renshaw cells and Ia inhibitory neurons [29]. Both classes of cell are known to project monosynaptically to motoneurons [40]–[45]. Putative Renshaw cells labeled with the virus usually formed small clusters at the most ventral edge of the MN pool (Fig. 6A–C), had cell bodies 10–20 µm in diameter and expressed the calcium binding protein calbindin-D28K (Fig. 6B; [29]). Consistent with the identity of these cells as Renshaw cells we found that some of their virally labeled, calbindin-immunoreactive neurites had swellings apposed to motoneuron somata. (Fig. 6A, C). When the TA muscle was injected alone we found 11.2±4.6 (n = 3 animals) virally-labeled putative Renshaw cells per mm of spinal cord. When both the TA and the GC muscles were injected the number of labeled putative Renshaw cells rose to 60.5±18.6 cells per mm (n = 4 animals; p<0.01).

Double tissue immunoprocessing revealed that some of the virally labeled interneurons in lamina VII also co-expressed the calcium binding protein parvalbumin (PV; Fig. 6D and E). These cells were found medially with respect to the motoneuron pool and had cell bodies 20–30 µm in diameter (Fig. 6, D, E). These parvalbumin expressing neurons may represent a part of the Ia inhibitory interneuron population as they were previously shown to be located in the ventral part of lamina VII dorsal to the area occupied by Renshaw cells [29].

**Figure 7:**
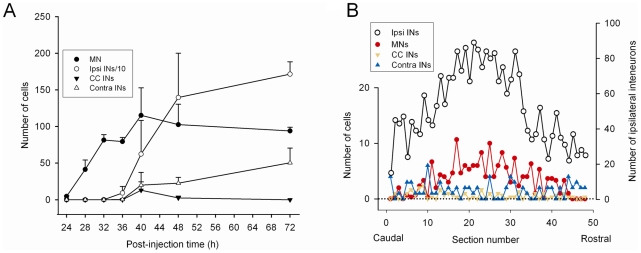
Distribution of PRV labelled neurons. **A**) Change in the number of PRV labelled neurons with different post-injection times obtained from 20 animals (2–5 animals per group). Labeled motoneurons were first detected at 24 hours following TA muscle injection and their numbers stabilized at 32–40 hours post-injection. Virally labelled, ipsilateral interneurons were first detected at 36 hours post-injection and their number stabilized 12 hours later (one-way ANOVA test, Holm-Sidak method for all pairwise multiple comparisons; p≤0.01 for single comparisons). A similar trend was observed in contralateral and central canal interneurons. Note the delay of approximately 8–12 hours between the rise in number of motoneurons and the rise in number of ipsilateral interneurons. Note that the number of ipsilateral interneurons is plotted as their original number divided by 10 (Ipsi INs/10). **B**) The distribution of virally labelled neurons along the rostro-caudal axis of the L4 and L5 segments of one animal injected at postnatal day 7 (P7) and killed 44 hours later. A color code was assigned to different classes of labelled neurons for clarity. Note that the right-hand y-axis is different for the ipsilateral interneurons (Ipsi INs).

We analyzed the change in the number of PRV-labeled neurons as a function of post-injection time in serial spinal cord sections obtained from 20 animals. Virally labeled ipsilateral interneurons were first detected in significant numbers at 36 hours post-injection and their number stabilized 12 hours later (one-way ANOVA test, Holm-Sidak method for pairwise multiple comparisons; p≤0.01 for single comparisons). The temporal separation between the rapid rise in the number of labeled motoneurons (filled circles, Fig. 7A) and ipsilateral interneurons (open circles, Fig. 7A) was about 12 h, suggesting that this is approximately the time taken for the viral propagation through a single synaptic relay. The change in number of virally labeled contra-lateral interneurons (contra INs) and central canal interneurons (CCINs) followed a similar trend (Fig. 7A).

As illustrated in 1 animal that survived for 44 h post-injection (Fig. 7B), the rostro-caudal distribution of virally labeled motoneurons and ipsilateral interneurons along the L4 and L5 spinal segments assumed a parallel, bell shaped form. This suggests that the majority of trans-neuronally labeled interneurons projecting either mono- or di-synaptically to motoneurons are restricted to the same segments as the motoneurons although it is clear that interneurons were also labeled more distantly from the motoneuron segments.

## Discussion

We have used PRV Bartha recombinants expressing fluorescent proteins [12] to identify neurons projecting to hindlimb motoneurons in the neonatal mouse spinal cord. Our results show that motoneurons are retrogradely labeled with the virus-conjugated fluorescent marker 24–32 h after viral injection into a hindlimb muscle, and that the labeled interneurons first appear in significant numbers at 36 h post-injection. These findings suggest that it takes approximately 12 h for the virus to infect last order interneurons projecting to motoneurons. Furthermore, different classes of interneurons known to connect monosynaptically with motoneurons are reliably and consistently labeled at 40 h post-injection together with other interneurons whose identity and function are currently unknown.

### Motoneuron Labeling

**Figure 8:**
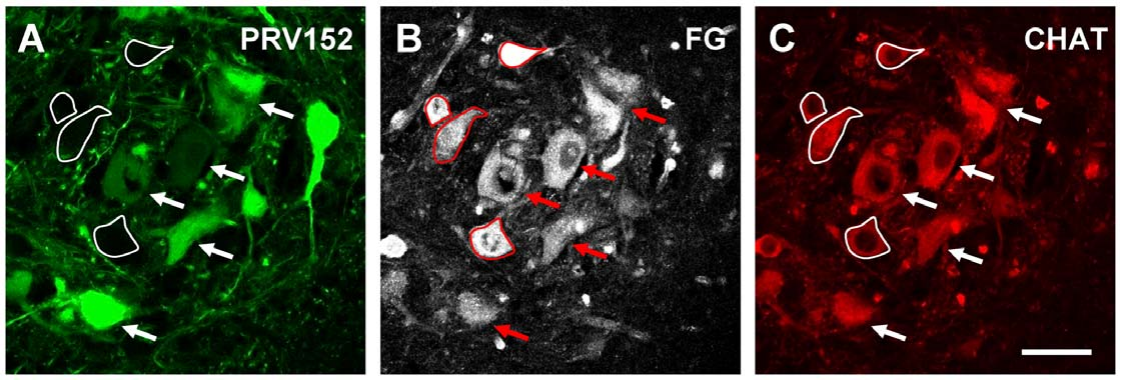
Preservation of the structural integrity of motoneurons 72 hours after injection of PRV-Bartha 152 into the GC muscle. **A**) Micrograph of the motor nucleus showing motoneurons (arrows) and other neurons labelled with PRV-Bartha 152, 72 hours after injection into the gastrocnemius muscle. Note that some of the motoneurons are not labelled with the virus (outlined) and their identity as motoneurons is established by the presence of fluorogold labelling (B) and CHAT immunocytochemistry (C). Scale bar in C is 50 µm.

At 24 h post-injection, ipsilateral TA motoneurons were the first spinal cells to express virus-conjugated fluorescence and their number increased over the next 8 hours before stabilizing. Because low levels of the virus may not have produced sufficient fluorescence to be detectable it is possible that the virus is present in motoneurons earlier than 24 hrs. The persistence of motoneuron labeling at 72 h (Fig. 8) suggests that the virus did not kill the cells during this period.

When the virus was injected into a muscle together with a retrograde marker, the viral labeling of motoneurons was often lower and less robust than that obtained with the retrograde label (data not shown). Similar findings were obtained with three different retrograde tracers (FG, Texas Red and TMRD) suggesting that the problem was not due to the particular tracer used but may have been the result of limitations in the capacity of the retrograde transport machinery in dual tracing paradigms [19].

We found that the smallest motoneurons were labeled first followed by larger ones. Although size alone cannot unequivocally distinguish α- from γ-motoneurons, γ-motoneurons likely comprise the majority of motoneurons with a surface area <500 µm^2^ at P8 [37]. Accordingly, because the motoneurons expressing the earliest viral labeling had surface areas <400 µm^2^ it seems reasonable to suppose that many are γ-motoneurons. It is possible that due to their small size γ-motoneurons may have been easier to fill with the viral progeny than the larger α-motoneurons that possess a more complex soma-dendritic tree and a greater internal volume. Alternatively, the virus may have a greater affinity for the terminals of γ- compared to α-motoneurons [19].

### Last order spinal neurons projecting to motoneurons are labeled between 36–48 h post-injection

Although a small number of labeled interneurons were observed at approximately 8 h after the first motoneurons were labeled, the number of labeled interneurons rose rapidly between 36–48 h post-injection (see Fig. 7A). Between 48–72 h the number of labeled interneurons increased more slowly. These data suggest that trans-neuronal transfer takes between 8 and 12 h, both for motoneuron to interneuron and then from interneuron to interneuron. It is difficult to compare these findings with those from other groups because no studies have systematically investigated the timing of viral labeling of spinal neurons following injection into hindlimb muscles. Kim and coworkers [20] examined the pattern of spinal labeling following PRV injection into the sciatic nerve of adult rats. At 48 h post-injection large diameter neurons, presumed to be motoneurons, were seen in the ventral horn. By three days post-injection, the number of labeled large diameter cells in lamina IX increased and labeled neurons appeared in laminae IV–VII. Similarly, Rotto-Percelay et al., [19] injected the virus into the medial gastrocnemius of adult rats but did not examine the spinal cord until 4 days post-injection, at which time both motoneurons and interneurons were labeled.

A more carefully timed study was made with injections of Bartha virus into the phrenic nerve of adult rats [8]. At 52 h post-injection, only phrenic motoneurons were labeled and labeled spinal interneurons first appeared between 52–71 h in lamina VII and IX. Unfortunately the precise time for motoneuron labeling was not reported in this study so it is not possible to estimate the transfer time for the virus from motoneurons into second order neurons. At 72 h bilateral labeling was observed. Another study by the same authors [9] examined the labeling pattern following viral injections into the tongue, again in the adult rat. In this case motoneurons were labeled by 32–36 h and second order neurons between 52–56 h suggesting a transfer time of 20 h. In contrast, another group, investigating viral injections into the tongue, found that motoneurons were labeled 52–56 h after the injection and the first interneurons appeared at 60 h [13].

Consistent with the idea that last order neurons were labeled between 36–40 h, we observed labeling in three populations of interneurons that are known to make monosynaptic connections with motoneurons. The first of these was the inhibitory Renshaw cell [29], [42], [46], [47] that was first labeled 40 h post-injection. The second was a class of cholinergic interneurons located near the central canal that appear to give rise to C-terminals on motoneurons [38], [39].

The third group includes Ia inhibitory interneurons (Fig. 4; Fig. 6D, E) that are found in a crescent formation in laminae V–VII dorsal and medial to the MN pool in the adult cat [42] but also in ventral lamina VII of the cat [48] and mice [29]. A distinct possibility exists that those found more dorsally with respect to the motoneuron pool may receive a stronger input from low threshold Ia afferents [43] while those located ventrally may be identified by a stronger input from calbindin-immunoreactive Renshaw cells [29]. Furthermore, interneurons that co-express various calcium binding proteins such calbindin-D28k and parvalbumin [29] and parvalbumin and calretinin [48] were also found in this region known to be populated with last order premotor interneurons [49].

Another group of neurons consistently expressing virus at 40 h postinjection was found in the contralateral lamina VIII (Fig. 5A, E), an area containing various groups of commissural interneurons [50], [51] some of which have been shown to project monosynaptically to contralateral motoneurons in the rodent spinal cord [22], [51]. Traditionally, these neurons have been implicated in mediation of crossed reflexes in the cat [52], [53] but also in the selection of different motor patterns [50], [51].

In addition to the above mentioned neuronal classes, neurons expressing the virus at 40 h postinjection were found in more dorsal laminae (Fig. 4, Fig. 5). While some of these neurons may belong to laminae V and VI interneurons subserving non-reciprocal inhibition of motoneurons [54] and/or to the recently defined group of inhibitory interneurons located in medial laminae V/VI [55], the identity and function of the majority remain to be determined.

Surprisingly, the number of PRV virally-labeled putative Renshaw cells was rather small when the TA muscle alone was injected and increased after both GC and TA were injected. One possibility to explain this data is that the number of Renshaw cells may vary with the muscle function i.e. may be greater in extensors vs. flexor muscles [56], [57]. Alternatively, a greater fraction of a common Renshaw pool may be activated if motor pools normally involved with stereotyped motor output are activated together [58] as mimicked in this study by injecting both tibialis anterior and gastrocnemius muscles.

### Mechanism of trans-neuronal labeling

The mechanisms responsible for the trans-neuronal transfer of PRV-Bartha are incompletely understood. Studies in hamsters and rats have shown that intraocular injection of the virus labels the hypothalamic suprachiasmatic nucleus by retrograde trans-synaptic transport through autonomic pathways [59], [60] although initially it was thought that labeling of the nucleus was mediated anterogradely through the projections of retinal ganglion cells [61]. In the present work, motoneurons and interneurons could have been labeled by anterograde transport of the virus from muscle afferents or recurrent motoneuron collaterals and at longer incubation times through anterograde transport from retrogradely labeled interneurons. We do not believe this to be a significant complication for several reasons. First, no viral labeling was found in the parvalbumin-immunoreactive muscle afferent axons (Fig. 2). Consistent with this result, Rotto-Percelay et al., [19] found no difference in viral labeling in the spinal cord between normal rats and those with dorsal rhizotomies. Secondly, if retrogradely labeled interneurons subsequently infected motoneurons anterogradely then we would have expected an increase in the number of motoneurons expressing fluorescence at the longest incubation times (72 h). However, the number of labeled motoneurons stabilized at 32 h and did not change significantly thereafter. Consistent with this view, it is now generally agreed that retrograde trans-synaptic transport is a major mechanism of trans-neuronal labeling with PRV-Bartha (for review see [62]).

However, whether or not this is the only mechanism of cell to cell viral transfer is not clear. It is known that mice infected with PRV Bartha exhibit neurological deficits ∼140 hrs after infection and die at ∼220 hours after infection [63]. It is, therefore, possible that infected motoneurons might die and release their contents, including the virus, into the extracellular space. As a result, it would not be possible to attribute the labeling pattern of interneurons to trans-synaptic labeling. However, we believe this is unlikely in the present experiments because the number of labeled motoneurons peaked at 32 h hours and remained constant thereafter. If lysis or death had occurred as a result of the viral infection, we would anticipate it to be manifest in motoneurons first because these cells were labeled longer than other neuron types. Moreover, motoneurons at 72 hours maintained their structural integrity without evidence of lysis of extracellular virus (Fig. 8). Furthermore, if such non-specific spread had occurred it would be difficult to explain how the earliest interneurons labeled by the virus included those known to be connected monosynaptically to motoneurons. In summary, while we cannot exclude motoneuronal damage and lysis at longer post-infection times our work suggests this is not prominent in the spinal cord before 72 hours post-infection.

Finally, a recent report has shown that cultured superior cervical ganglion neurons infected with PRV-Bartha 152 exhibit abnormal firing rates and develop low resistance junctions capable of passing large molecular weight dextrans [64]. Clearly, if such changes are present in the spinal cord it will be crucial to establish when they occur and how they might compromise neural function. McCarthy et al., [64] proposed that the changes they observed in neurons infected with PRV-Bartha 152 might be responsible for the late neurological abnormalities exhibited by the infected mice. However, these appear at ∼140 hours post-infection well after the latest times we examined in the present work.

### Concluding remarks

We analyzed the circuits that control hindlimb motoneurons using fluorescent PRV Bartha recombinants. By taking advantage of the viral self-amplifying properties and transsynaptic propagation, we defined the time course of the viral spread in the neonatal mouse spinal cord and visualized interneurons potentially involved in direct modulation of motor neurons. Viral markers, unlike other available markers, are capable of identifying collectively the neurons directly connected to a cell group of interest. Furthermore, the latest derivatives of PRV Bartha can be conditionally expressed only in certain types of neurons [65] or, by means of color coding that reports which neurons have been infected (rainbow virus [66]). Thus, when combined with immunocytochemistry and/or intracellular recording, the use of these markers should enable a more comprehensive analysis of synaptic connectivity within motor circuits than is currently possible. Of course, one concern is that the infected neurons may not be healthy and/or their physiological properties may be altered by the presence of the virus, particularly at prolonged incubation times. However, it has been demonstrated [11] that infection by PRV152 does not interfere with normal synaptic activity in brain slices and functionally related neurons can be analyzed in the context of their local synaptic networks for up to 5 days. Finally, it has recently been shown that PRV-Bartha virus can be combined with the calcium sensitive protein G-CaMP2 to allow imaging of synaptically connected neurons [67].

In future experiments, it should be possible to visually target the labeled interneurons for intracellular recording. This would enable confirmation of a last order connection with motoneurons and would facilitate the targeting of known cell types (e.g Renshaw cells, Ia inhibitory interneurons). In addition, the labeling pattern at 40 hours revealed many cell types whose identity is currently unknown. By comparing the labeling pattern with that of transcription factor expression it may be possible to identify novel last-order neurons. Such physiological studies would also allow us to establish if the infected neurons are healthy or if their physiological properties are altered by the presence of the virus. Finally, the use of the viral markers may be advantageous not only in elucidating organization of the intact lumbar neuronal networks but also in evaluating changes that these networks may undergo after spinal cord injury.
